# Enhancing LPG Adoption in Ghana (ELAG): A Trial Testing Policy-Relevant Interventions to Increase Sustained Use of Clean Fuels

**DOI:** 10.3390/su13042213

**Published:** 2021-02-19

**Authors:** Daniel Carrión, Rebecca Prah, Theresa Tawiah, Oscar Agyei, Mieks Twumasi, Mohammed Mujtaba, Darby Jack, Kwaku Poku Asante

**Affiliations:** 1 Department of Environmental Medicine and Public Health, Icahn School of Medicine at Mount Sinai, New York, NY 10029, USA; 2 Kintampo Health Research Centre, Kintampo, Ghana; 3 Department of Environmental Health Sciences, Mailman School of Public Health, Columbia University, New York, NY 10032, USA

**Keywords:** household energy transitions, solid fuel, LPG, sustained use, sustainable development, intervention trial, behavior change

## Abstract

Rural Ghanaians rely on solid biomass fuels for their cooking. National efforts to promote the Sustainable Development Goals include the Rural Liquefied Petroleum Gas Promotion Program (RLP), which freely distributes LPG stoves, but evaluations have demonstrated low sustained use among recipients. Our study objective was to assess if cheap and scalable add-on interventions could increase sustained use of LPG stoves under the RLP scheme. We replicated RLP conditions among participants in 27 communities in Kintampo, Ghana, but cluster-randomized them to four add-on interventions: a behavioral intervention, fuel delivery service, combined intervention, or control. We reported on the final 6 months of a 12-month follow-up for participants (*n* = 778). Results demonstrated increased use for each intervention, but magnitudes were small. The direct delivery intervention induced the largest increase: 280 min over 6 months (*p* < 0.001), ∼1.5 min per day. Self-reported refills (a secondary outcome), support increased use for the dual intervention arm (IRR = 2.2, *p* = 0.026). Past literature demonstrates that recipients of clean cookstoves rarely achieve sustained use of the technologies. While these results are statistically significant, we interpret them as null given the implied persistent reliance on solid fuels. Future research should investigate if fuel subsidies would increase sustained use since current LPG promotion activities do not.

## 1. Introduction

Combustion of solid fuels in open fires is the dominant form of cooking and heating for 3 billion people worldwide [1], resulting in household air pollution and related morbidity and mortality [2]. In Ghana, solid fuel use is ubiquitous in rural areas [3]. To address health and deforestation concerns, and to meet national energy policy targets based on the Sustainable Development Goals [4,5], the Ghanaian government initiated the Rural Liquefied Petroleum Gas (LPG) Promotion Program (RLP). The RLP aimed to increase LPG use by distributing free LPG stoves in rural areas [6]. Efforts to promote LPG and other clean fuels are underway in many low-to-middle-income countries worldwide [7,8]. Evaluations of the program have found low levels of sustained LPG stove use among stove recipients, both in Ghana and elsewhere [9,10]. Therefore, the RLP and similar efforts to promote clean fuels may be helped by low-cost and scalable add-on interventions that increase sustained use among stove recipients.

It has long been observed that receiving or purchasing a new cookstove (adoption) does not mean recipients will sustain use of the stove over time [11–13], which we have also observed in Ghana [9,10,14]. Research has found that there are many determinants of cookstove adoption and sustained use, broadly categorized into: (1) household/community characteristics, (2) access issues, and (3) knowledge, perceptions, and attitudes [12,15–18]. Issues that drive adoption likely differ to those that drive sustained use, but fewer studies have focused on determinants of sustained use [13,19]. Cookstove adoption and sustained use research span many disciplines and methods [20–24], but the field has largely employed qualitative, retrospective, or observational study designs. These studies are informative, but are more vulnerable to confounding, selection biases, or their findings may not be generalizable. Therefore, they may not provide sufficient evidence on which to base large-scale policy interventions. Controlled trials can address these limitations through randomization of policy-relevant interventions, but few studies have used these designs [25,26].

We conducted a cluster-randomized factorial intervention trial called Enhancing LPG Adoption in Ghana (ELAG) [18], to provide evidence on potential sustained use interventions in Ghana. We replicated the conditions of the RLP by distributing free LPG stoves to participants in rural communities. However, we added two interventions to address potential barriers to sustained use. These interventions were: (1) a behavioral change intervention using the Risks, Attitudes, Norms, Abilities, and Self-Maintenance (RANAS) [27] model, and (2) an access intervention to improve the ease of refueling LPG cylinders. The factorial design also allowed us to evaluate the interaction of these two interventions. We then tracked participant stove use for one year via stove use monitors (SUMs) and considered sustained use as the average use in the last six months of the study.

## 2. Methods

### 2.1. Study Setting

The study took place in Kintampo North Municipality and Kintampo South District in the Bono East Region of Ghana. This is a mostly rural area (population 212,198) [28], and households predominantly use wood or charcoal for their cooking needs [29]. There are two seasons, wet and dry. Most cooking takes place outdoors in the dry season and in enclosed or covered kitchen areas in the wet season. Enrollment was on a rolling basis, starting in June 2017, and the study ended in October 2018.

### 2.2. Study Participants and Ethical Approvals

ELAG participants were limited to women and households who had participated in a preceding health study [30], and who: (1) had never received an LPG stove and (2) still resided in the KHRC study region (Supplemental Figure S1). We focus on women as our main enrollees because they were the main participants of the preceding study, but we encouraged participation from men when they were available. The study was registered with clinicaltrials.gov (NCT03352830).

### 2.3. Study Design

#### 2.3.1. Interventions

All participants received an LPG stove and two 14.5 kg cylinders at no cost to them. This study employed a factorial intervention design with two separate interventions: a health promotion intervention following the RANAS model (Table 1), and an access intervention, where some participants received on-demand direct delivery of LPG refills from local taxi drivers. Interventions are detailed in Appendix A and in our protocol [18]. We also employed a combined intervention. This resulted in four arms: (1) Control arm, (2) RANAS health promotion, (3) direct delivery, and (4) dual intervention (RANAS and delivery) recipients. All participants received a free first cylinder of LPG fuel with their LPG stove, but subsequent refills were at their own expense. The RANAS arms included regular follow up visits by local community-based surveillance volunteers (CBSVs; see Appendix A). Participants were monitored for one year after receiving their stove.

#### 2.3.2. Cluster Randomization

We used covariate-constrained randomization with prognostic covariates to assign clusters to study arms [31,32]. We chose this approach because of its efficiency in dealing with imbalance in cluster designs. Prognostic covariates were balanced (Table S1), and then randomization was conducted by an independent epidemiologist using the ccrand procedure in Stata. Allocation was not revealed to field staff until after baseline data collection.

### 2.4. Data Collection and Management

#### 2.4.1. Baseline Data

Baseline demographic and socioeconomic status surveys were administered by field staff. We constructed a household asset index using a principal components analysis of variables, including type of housing materials, type of toilet facility, primary water source, type of home ownership, ownership of livestock animals, and ownership of consumer durables [33]. Pre-/post-tests of the RANAS model behavioral factors were administered to assess changes in participants’ knowledge, perceptions, or attitudes regarding household air pollution and/or LPG stoves.

#### 2.4.2. Stove Use Monitoring

The principal outcome was average weekly cooking time (minutes per week) using an LPG stove during the last 6 months of the study as a proxy for sustained use. Stove use was measured via stove use monitors (SUMs). Field staff visited households every two weeks to download the data. We experienced differential data missingness by arm (Table S2), with higher missing data rates in the direct delivery and dual intervention arms. Missing data were higher among younger participants in smaller households, which we believe resulted in a downward bias (Table S3). Consequently, we employed an imputation model using LPG cylinder-weighing data to account for missing observations. The primary inputs in the imputation model were bi-weekly estimates of fuel use obtained by cylinder-weighing. See Appendix A for additional detail. We interpreted the imputed dataset as our main results of interest.

#### 2.4.3. Other Measures of Use

Field staff assessed two other measures of use during biweekly visits: (1) the weight of LPG cylinders (EatSmart Precision Voyager Luggage scale) and (2) a survey asking whether the household had refilled cylinders since the last visit.

### 2.5. Data Analyses

Our primary outcome was summed stove use over the final six months of observation, comparing each intervention arms to the control. Response data were not normally distributed, so we conducted pairwise Wilcox rank sum tests. The secondary analysis was to compute and compare unadjusted incidence rate ratios from self-reported refills by arm as a function of observation time (person-weeks of fieldworker visits). We assessed potential differences in subgroup effect by modeling interactions between study arms and sociodemographic variables of interest. We performed a log-linear regression with cluster-robust standard errors by village to account for potential clustering of observations.

## 3. Results

### 3.1. Trial Profile and Participant Characteristics

**Figure 1.**
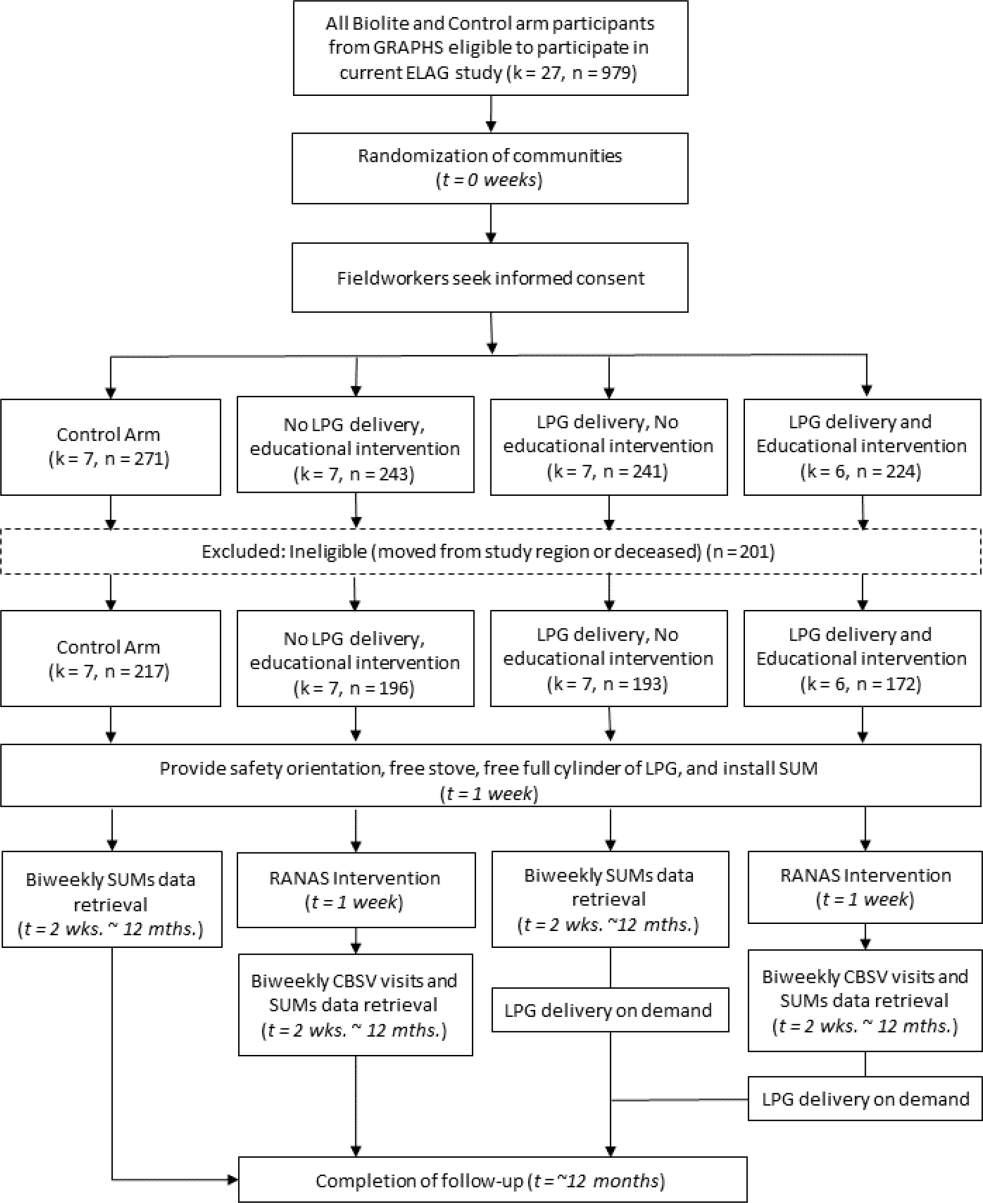
Trial design and profile.

There were 979 eligible households in our study (Figure 1). Communities were cluster-randomized before enrollment, yielding seven communities in the control arm (*n* = 271), seven in the direct delivery arm (*n* = 243), seven in the RANAS education arm (*n* = 241), and six in the dual intervention (*n* = 224). Overall, 201 were either ineligible due to leaving the study area or being deceased. The final sample was 778. There was no attrition after enrollment.

Participants were similar in most respects (Table 2). On average, participating women were slightly above 30 years old, mostly Christian, lived in households of 6–10 people, and had approximately 6 years of formal education.

## 3.2. Effect of Intervention on RANAS Factors

**Figure 2.**
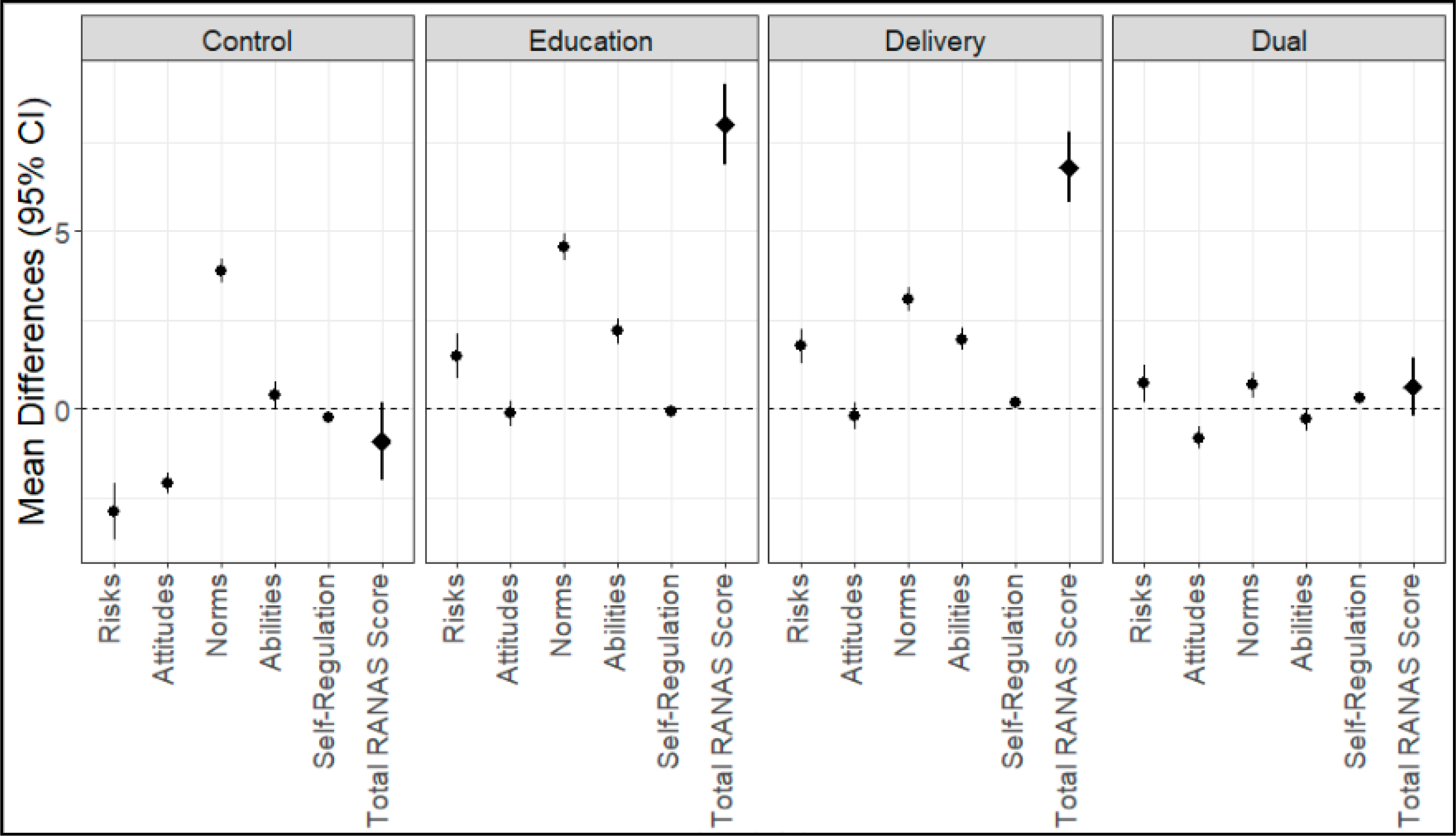
Mean differences, in RANAS behavioral factors (circle) and overall score (diamond), for the pre- and post-tests by arm of Enhancing LPG Adoption in Ghana (ELAG). Scoring is positive when oriented toward behavior change, and test is out of 105 points. 95% confidence intervals from paired *t*-tests, *n* = 778.

We administered an evaluation on the five RANAS behavioral factors at baseline and at study closeout. This evaluation was designed to assess knowledge and attitudes towards household air pollution, traditional, and clean cooking fuels. The maximum score on the evaluation was 105 points. Scores had a mean of 86.7 points at baseline and 90.3 at study closeout. We present mean differences by arm (Figure 2). The total RANAS score did not change significantly for the control arm (−0.91 points, 95% CI: −1.99–0.17) or the dual intervention arm (0.61 points, 95% CI: −0.2–1.42). However, the total RANAS score did increase for the RANAS education arm by 8 points (95% CI: 6.86–9.14), a 9.3% increase from the baseline. The direct delivery arm increased by 6.78 points (95% CI: 5.79–7.78), a 7.8% increase from the baseline. A notable difference within the specific RANAS factors was a decrease in the risks score for the control arm, while other arms exhibited small increases. Intervention tracking data showed a lower number of CBSV follow-up visits in the dual intervention arm rather than the RANAS education arm. CBSV visits were associated with higher RANAS scores (see Appendix A and Table S4).

### 3.3. Effect of Intervention on Sustained Use

Our imputed results showed that all intervention arms had a statistically significant increase in sustained use (Table 3). The RANAS education arm had the smallest increase (*p* < 0.001) and the direct delivery arm had the largest (*p* < 0.001). This represented a median increase of 280 min over 6 months, or ∼11 additional minutes per week compared to the control arm.

We assessed the consistency of our results by examining alternate measures of sustained use (Table 4). We used the self-reported number of refills as an alternate event of interest. We found that the dual intervention arm had a higher incidence of refills per weeks of visits (IRR: 2.2, *p* = 0.026) when compared to the control arm over the last six months of the study. Over the full year of follow up, we found that all three intervention arms had a higher number of refills compared to the control arm. The highest incidence of refills again took place in the dual intervention arm, with an incidence rate ratio of 2.38 (*p* = 0.002) when compared to the control arm. A higher incidence of refills also emerged for the RANAS education arm (IRR: 2.01, *p* = 0.002) and the direct delivery arm (IRR: 1.71, *p* = 0.037). LPG refueling prices are provided for reference (Table S5).

### 3.4. Trends and Heterogeneity of Treatment Effect

We visualized use over the study period to understand potential temporal trends relative to study enrollment (Supplemental Figure S2). There was no evidence of heterogeneity of treatment effect by participant socio-demographic characteristics (Table S6). However, power calculations were not conducted for subgroup analysis, which typically requires higher sample size to detect effect modification.

## 4. Discussion

ELAG was a cluster-randomized controlled intervention trial to estimate differences in the sustained use of clean cookstoves by two scalable and low-cost interventions, both separately and in combination. We found that the RANAS educational intervention led to increases in knowledge and attitudes towards behavior change. However, we also observed increases in knowledge and attitudes in the direct delivery arm and no change in the dual intervention arm. While there was notable SUMs data loss, our imputed results showed that all three intervention arms yielded up to 280 min more use than the control arm over the sustained use period. While these results were statistically significant, stove use was low overall and the small differences indicate persistent stove stacking with traditional stoves. Thus, our overarching conclusion is that none of our interventions induced meaningful additional LPG use. While stove stacking is a common phenomenon [34], in this context, we are concerned with the persistent use of biomass fuels because it is associated with higher levels of air pollution exposure [29] and related health conditions [35]. The results of this study, limited sustained use among stove recipients, are consistent with retrospective and observational studies in this field [11].

This study had notable limitations. Data loss from our SUMs likely induced downward bias from differentially missing observations. We account for this with an imputation model using our other fuel use measures, and those results demonstrated associations in line with our a priori hypotheses. Another potential limitation is that our intervention does not control for the potential role of fuel price, which could have substantial influence on use. However, fuel price did not vary substantially, and our goal was to see if the interventions would increase sustained use under the existing policy landscape. Finally, our secondary outcome (self-reported refills) may be vulnerable to social desirability bias. If so, we are unable to assess the potential direction or magnitude of the bias, since participants in each arm knew that our interest was in sustained use of the stoves. However, weighing the cylinders and measuring stove use with sensors offered physical measurements that are unlikely to be influenced by desirability.

Our study has many strengths. The study participants are part of a long-standing cohort for which we have considerable data. We then designed two context-specific and scalable interventions that could directly inform Ghanaian energy policy and tested them using a rigorous randomized design. For many years, the Ghanaian government has been trying to increase LPG uptake in rural areas, largely through the RLP [9]. The RANAS intervention was chosen for its successes in modifying knowledge and perceptions for water, sanitation and health initiatives in Sub-Saharan Africa [36–38] and the direct delivery model was to simulate a more accessible energy distribution network in Ghana. Furthermore, while we are currently reporting on an intention-to-treat analysis, we have also collected rich data on fuel access (price and distance), household gender dynamics, and household characteristics [18,39]. Future secondary analyses will assess these as probable mediating factors of sustained use.

These results have important implications for Ghanaian energy policy and sustainable development initiatives because the national government is currently overhauling its LPG energy policies [40] and can consider other ways to increase LPG adoption and sustained use in the process. Many studies suggest that free (or low-cost) clean cookstoves may not adequately incentivize sustained use [41–43], which this study corroborates with data from rural Ghana. The RLP is essentially a subsidy on stoves, but ongoing research in northern Ghana suggests that willingness to pay for stoves is actually higher than market price and willingness to pay for LPG is below market price [44]. If so, this would suggest that the current subsidy structure in the RLP should be placed on fuel. Our research team is conducting a similar study in Kintampo to assess how demand for LPG is influenced by price changes. Although Ghana’s current policy does not include subsidy on LPG prices, the findings of this study could provide evidence to guide policies on LPG in Ghana. Other countries around the world successfully subsidize clean fuels to support sustained use [45]. Therefore, the available evidence suggests that Ghana should carefully consider subsidy frameworks that include LPG fuel.

## 5. Conclusions

This study contributes to the literature on the sustained use of clean cookstoves by employing novel interventions and a policy-relevant randomized design. We replicated the conditions of the Ghanaian RLP by providing participants with free LPG stoves, but not subsidizing LPG fuel refills. Then, we used a randomized design and showed that the RANAS health behavior model and a direct delivery intervention may offer marginal gains in sustained use of the cookstoves. These findings indicate that these interventions are insufficient to promote sustained use of LPG under the current RLP strategy. We recommend consideration of other subsidies, such as a fuel subsidy, to enhance sustained use in these areas. Only by decreasing traditional stove use can we appreciate the health and ecological benefits of clean cooking.

## Supplementary Material

Supplementary material Figure S1: Map of the study region, Table S1: Prognostic covariates included in cluster-randomization procedure, Table S2: Missingness by arm, Table S3: Characteristics of participants with less than one month’s worth of data in the latter six-month period, Table S4: Univariable linear regressions of participant and intervention characteristics on total RANAS score change from the pre to post tests, Figure S2: Time series of use over the entire study period, Table S5: Cost of a 14.5 kg cylinder refill at local refilling station, Table S6: Sub-group analysis of treatment effects.

## Figures and Tables

**Table 1.** Overview of Risks, Attitudes, Norms, Abilities, and Self-Maintenance (RANAS) intervention, delivered by local community members and field staff.

**Risks**	Education on the health impacts of household air pollution (HAP) exposure, and potential benefits of mitigation.
**Attitudes**	Discussion of non-health benefits of clean cooking, including time savings, safety, and cleaner pots/utensils.
**Norms**	Convening intervention with other participants in a public setting, prompting collective commitment to using liquefied petroleum gas, discussing government policies towards clean cooking.
**Abilities**	Financial orientation—strategies to save for LPG refills. Identifying all refill locations. Having a peer LPG adopter: do a cooking demonstration, discuss a time when they could not refill due to financial or logistical constraints.
**Self-Regulation**	Weekly follow-up visits from a community member contracted by the study.

**Table 2.** Baseline characteristics by study arm.

	Control (*n* = 217)	Education (*n* = 196)	Delivery (*n* = 193)	Dual (*n* = 172)	Total (*n* = 778)

**Participant’s Age**					
Mean (SD)	31.1 (7.1)	31.8 (7.7)	31.0 (7.1)	32.0 (7.4)	31.4 (7.3)

**Ethnicity**					
Akan	78 (35.9%)	17 (8.7%)	41 (21.2%)	41 (23.8%)	177 (22.8%)
Grushi	8 (3.7%)	25 (12.8%)	22 (11.4%)	6 (3.5%)	61 (7.8%)
Dagarti	54 (24.9%)	74 (37.8%)	47 (24.4%)	37(21.5%)	212 (27.2%)
Mo	6 (2.8%)	53 (27.0%)	41 (21.2%)	16 (9.3%)	116 (14.9%)
Konkomba	44 (20.3%)	9 (4.6%)	18 (9.3%)	29 (16.9%)	100 (12.9%)
Other	27 (12.4%)	18 (9.2%)	24 (12.4%)	43 (25.0%)	112 (14.4%)

**Religion**					
Christian	153 (70.5%)	143 (73.0%)	137(71.0%)	118 (68.6%)	551 (70.8%)
Non-Christian	64 (29.5%)	53 (27.0%)	56 (29.0%)	54 (31.4%)	227 (29.2%)

**Household Size**					
2–5 persons	75 (34.6%)	53 (27.0%)	74 (38.3%)	68 (39.5%)	270 (34.7%)
6–10 persons	116 (53.5%)	112 (57.1%)	98 (50.8%)	89 (51.7%)	415 (53.3%)
More than 10 persons	26 (12.0%)	31 (15.8%)	21 (10.9%)	15 (8.7%)	93 (12.0%)

**Participant’s Profession**					
Secretarial/Professional	2 (0.9%)	2(1.0%)	0 (0.0%)	1 (0.6%)	5 (0.6%)
Trader	68 (31.4%)	56 (28.6%)	65 (33.7%)	44 (25.6%)	233 (29.9%)
Seamstress	7 (3.2%)	12 (6.1%)	16 (8.3%)	8 (4.7%)	43 (5.6%)
Farmer	114 (52.5%)	85 (43.4%)	78 (40.4%)	94 (54.6%)	371 (47.7%)
No formal employment	26 (12.0%)	41 (20.9%)	34 (17.6%)	25 (14.5%)	126 (16.2%)

**Participant’s Education (years)**					
Mean (SD)	6.5 (5.7)	6.4 (5.8)	7.6 (5.6)	6.3 (5.7)	6.7 (5.7)

**Wealth Index quintile**					
1 (very poor)	49 (22.6%)	43 (21.9%)	35 (18.1%)	27 (15.7%)	154 (19.8%)
2	49 (22.6%)	43 (21.9%)	31 (16.1%)	34 (19.8%)	157 (20.2%)
3	43 (19.8%)	37 (18.9%)	41 (21.2%)	37(21.5%)	158 (20.3%)
4	40 (18.4%)	37 (18.9%)	40 (20.7%)	38 (22.1%)	155 (19.9%)
5 (least poor)	36 (16.6%)	36 (18.4%)	46 (23.8%)	36 (20.9%)	154 (19.8%)

**Table 3.** Comparison of median and interquartile range of stove use (in minutes) by arm of study in the last six months of the observation period. *p* values produced from Wilcox rank sum tests. *n* = 778.

	Arm	Median (IQR)	*p* Value

Results without imputation	Control	120 (10–430)	Reference
	Education	160 (0–480)	0.668
	Delivery	0 (0–90)	<0.0101
	Dual	0 (0–110)	<0.000

Results with Imputation	Control	320 (170–560)	Reference
	Education	380 (280–670)	<0.001
	Delivery	600 (470–750)	<0.000
	Dual	580 (460–680)	<0.000

**Table 4.** Results from secondary measure of use: self-reported refills during bi-weekly fieldworker visits. Analysis for the last 6 months of the study period and full year of follow up. Incidence rate calculated with total refills and surveillance time (household visit weeks). *p* value calculated with Fisher’s test. Statistically significant values in bold. Households = number of unique households that refilled their cylinders.

		Biweekly Visits (Last 6 Months)						Biweekly Visits (Full Year)		

Arm	Refills	Households	Visit Weeks	Incidence Rate Ratio	*p* Value	Refills	Households	Visit Weeks	Incidence Rate Ratio	*p* Value

Control	17	14	1705	Reference		29	23	3428	Reference	
Education	27	26	1676	1.62	0.131	55	44	3236	2.01	0.002
Delivery	12	12	1045	1.15	0.705	33	27	2269	1.71	0.037
Dual	15	12	683	2.2	0.026	27	23	1338	2.38	0.002

## Data Availability

An anonymized/limited dataset may be made available on request due to ethical and privacy concerns of human subjects’ research.

## Appendix A.

### Appendix A.1. Methods

#### Appendix A.1.1. Study Participants and Inclusion Criteria

ELAG participants were a subset of the Ghana Randomized Air Pollution and Health Study (GRAPHS), which was a cluster-randomized controlled trial assessing the impacts of a stove intervention on childhood pneumonia and birthweight [30]. There were three arms, a control (3-stone fire) arm, an improved cookstove (BioLite; Brooklyn, NY, USA), and an LPG stove. ELAG participants were limited to those enrolled in the original GRAPHS cohort and who: (1) were originally randomized to the BioLite or control arms of the study and (2) still resided in the KHRC study region (Supplemental Figure S1).

#### Appendix A.1.2. Health Promotion Intervention

The Risks, Attitudes, Norms, Abilities, and Self-Maintenance (RANAS) model was selected to design a clean cookstove behavioral change intervention [27]. The RANAS model was first used in the water, sanitation, and hygiene domain. However, we recognized its potential application for clean cookstove adoption due to its emphasis on behavior change involving new technologies and its successful application in sub-Saharan Africa. The core assumption that underpins the RANAS model is that each of these five behavioral factors are necessary, but each alone is insufficient to promote behavior change. After baseline data collection, ELAG households were convened for LPG stove distribution and (if relevant) the behavioral change intervention. A research team member from KHRC and a peer-adopter collaborated to deliver the intervention. The peer-adopter was a participant from a GRAPHS LPG community who has sustained use of LPG after study conclusion.

A series of activities were undertaken to address each RANAS behavioral factor (Table 1). Details of the intervention have been published previously [18]. First, we explained the health risks associated with exposure to household air pollution. For attitudinal factors, we discussed perceptions of time, money, and effort associated with the behavior change, and the benefits of the new behavior. Normative factors were addressed via the establishment of group-level and individual-level expectations, i.e., the group delivery of the intervention, in the presence of peers and community leaders. The LPG peer adopter was also asked to provide a testimonial regarding their experiences using LPG and overall appreciation of the technology. Ability-related behavioral factors entailed a food demonstration by the peer-adopter where they cooked a typical meal on the LPG stove. We also designed and provided a financial literacy orientation so that participants would consider saving for future LPG purchases. Participants were provided with money-saving boxes to encourage saving towards LPG refill. Self-regulation factors refer to continued orientation to the desired behavior, given that personal and broader circumstances are oriented towards traditional cookstove use. ELAG addressed this factor by contracting and training community-based surveillance volunteers (CBSVs) who visited participants weekly to discuss the potential barriers to sustained use and brainstorm possible solutions. The CBSVs are community liaisons for the longstanding Kintampo Health and Demographic Surveillance system [46].

#### Appendix A.1.3. Access Intervention

Energy access research underscores the importance of the “last mile” (or, more realistically in rural Ghana, last 30 km) of LPG delivery/accessibility [14]. Few studies have tested how much these logistical barriers impact user demand [47]. To directly test the extent to which transportation costs limit LPG use, the ELAG access intervention employed community-based taxi drivers to refill LPG cylinders for participants. We contracted and paid the drivers for the delivery services, but participants were responsible for the cost of the refill.

#### Appendix A.1.4. Stove Use Monitoring

Stove use monitors were Maxim iButton temperature loggers, programmed to record temperature every ten minutes [48]. Downloads were conducted with Thermodata data downloaders (Thermodata, Eight Mile Plains, QLD, Australia). Temperature data were transformed into cooking time using the AnomalyDetection package in R [49] which, when applied to temperature data, detects events that deviate from the ambient diurnal pattern. We only considered positive slope anomalies and those above 35 °C as cooking time.

#### Appendix A.1.5. Missing Stove Use Data Imputation

Gathering complete stove use time series proved to be challenging. Major issues included SUMs failures (due to high temperature or moisture exposure), Thermodata downloader breakage, and missed downloads due to participants being away from home. Because LPG stoves are typically stored indoors, if a participant was not home during a field visit, we were unable to download their stove use data. While we obtained some data on all participants, the amount of missingness was differential by study arm, with greater missingness in the direct delivery and dual intervention arms of the study (Tables S1 and S2). To address this limitation, we leveraged the biweekly cylinder-weighing data to create a predictive model of weekly stove use using the times we had data overlap. Differences in biweekly weights were calculated and linearly interpolated to provide a weekly estimate. That estimate was combined with other variables, specifically: arm of study, household size, participant’s education, ethnicity, season, week of study, and asset index. We tested several regression types, and found that an ordinary least squares model offered the smallest mean absolute error in a 10-fold cross validation, with a 10% holdout as participants rather than observations. Predictions were rounded to the nearest 10-min increment to match stove use monitoring data. We repeated the primary analyses with observed values and the predicted estimates when the observed values were missing.

#### Appendix A.1.6. Baseline Data Collection

We also assessed the potential role of gender dynamics on sustained use. An intrahousehold relationship score was tabulated from a validated questionnaire probing relationship quality [50]. The questionnaire entails direct questions about financial decisions in the home, along with other relationship dynamics.

#### Appendix A.1.7. Statistical Environment

All analyses were conducted using the R Statistical Programming Language, version 3.5.1. We used the tidyverse packages for data manipulation and the plm package for linear pooled regression modeling.

### Appendix A.2. Results

#### CBSV Visits and RANAS Pre/Post Test

There was an average of 3 visits per participant in the dual intervention arm (634 total visits) and 8 visits per participant in the RANAS education arm (1846 total visits). A regression of intervention and participant characteristics found that each CBSV visit was associated with a 0.1-point increase (*p* = 0.02) in RANAS post test scores among participants.
