# Supplementary material for: Enhancing LPG Adoption in Ghana (ELAG): A Trial Testing Policy-Relevant Interventions to Increase Sustained Use of Clean Fuels
Source: Sustainability. Author manuscript; Available in PMC 2021 Nov 10. (PMC8580155; doi:10.3390/su13042213)
Supplement: Supplementary material — Figure S1: Map of the study region, Table S1: Prognostic covariates included in cluster-randomization procedure, Table S2: Missingness by arm, Table S3: Characteristics of participants with less than one month’s worth of data in the latter six-month period, Table S4: Univariable linear regressions of participant and intervention characteristics on total RANAS score change from the pre to post tests, Figure S2: Time series of use over the entire study period, Table S5: Cost of a 14.5 kg cylinder refill at local refilling station, Table S6: Sub-group analysis of treatment effects. [file NIHMS1730609-supplement-Supplementary_material.pdf]

Enhancing LPG Adoption in Ghana (ELAG): A trial testing policy-relevant interventions to increase sustained use of clean fuels

Supplementary materials

Carrión, et al.

Figure S1: Map of the study region.

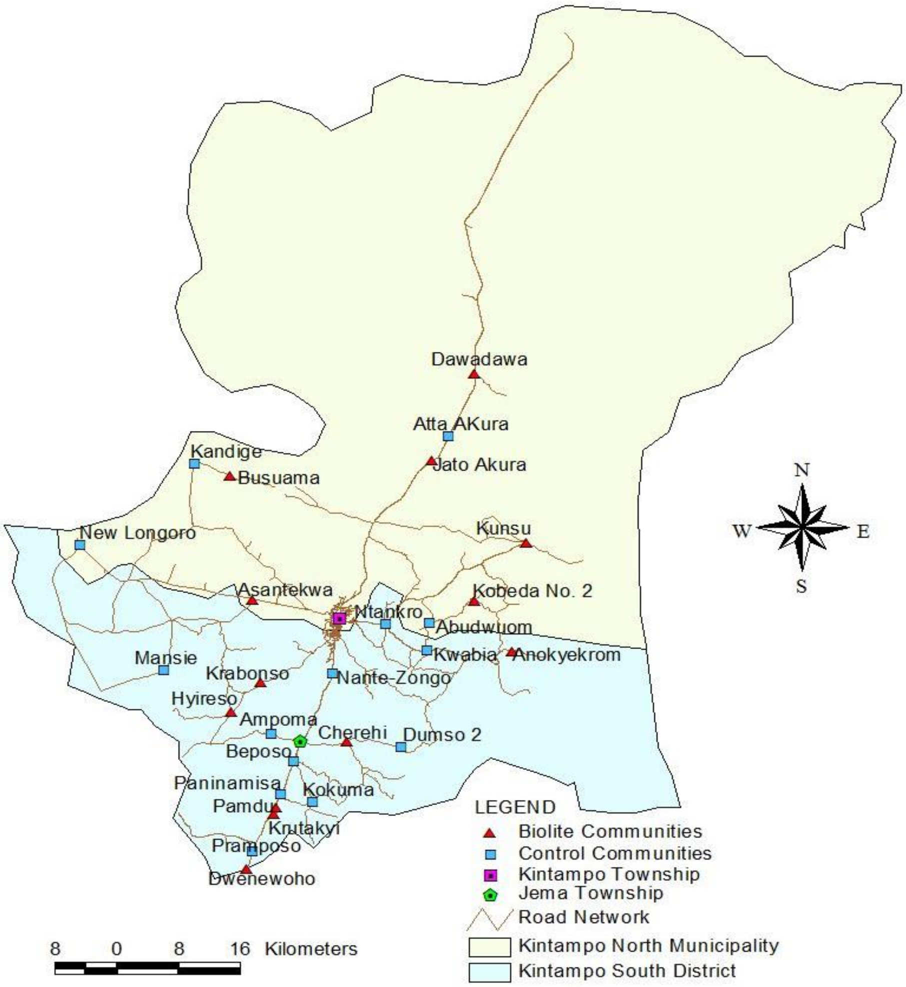

Table S1: Prognostic covariates included in cluster-randomization procedure.

**Variable**	**Rationale**
Community Asset Index	Studies have shown that differential access to resources can be predictive in the uptake of new cookstove technologies [41,42].
Average Household Size	Studies have shown that household size can be predictive in the uptake of new cookstove technologies [41,42].
Distance to Refueling Station	Participant communities are scattered throughout the region at varying distances from the refueling center. Further distances are likely a deterrent to refuel for non-Agent delivery households, **Supplemental Figure 1**.
Households per cluster	To ensure roughly equal number of participating households per arm.

Table S2: Missingness by arm. Participants for whom there are at least 30 days' worth of stove use monitoring data.

	**Control**	**RANAS Education**	**Direct Delivery**	**Dual**
**Participants with 30+ days of observations**	201	184	155	140
**Total enrolled**	217	196	193	172
**Percent for main analysis**	92.6%	93.9%	80.3%	81.4%

Table S3: Characteristics of participants with less than one month's worth of data in the latter six-month period.

	Missing (N=98)	Not Missing (N=680)	Total (N=778)	p value
**Participant's Age**				0.049
Mean (SD)	30.1 (7.2)	31.6 (7.3)	31.4 (7.3)	
**Ethnicity**				0.312
Akan	27 (27.6%)	150 (22.1%)	177 (22.8%)	
Other	15 (15.3%)	97 (14.3%)	112 (14.4%)	
Grushi	10 (10.2%)	51 (7.5%)	61 (7.8%)	
Dagarti	28 (28.6%)	184 (27.1%)	212 (27.2%)	
Mo	11 (11.2%)	105 (15.4%)	116 (14.9%)	
Konkomba	7 (7.1%)	93 (13.7%)	100 (12.9%)	
**Religion**				0.705
Christian	71 (72.4%)	480 (70.6%)	551 (70.8%)	
Non-Christian	27 (27.6%)	200 (29.4%)	227 (29.2%)	
**Household size**				< 0.001
2-5 persons	53 (54.1%)	217 (31.9%)	270 (34.7%)	
6-10 persons	41 (41.8%)	374 (55.0%)	415 (53.3%)	
More than 10 persons	4 (4.1%)	89 (13.1%)	93 (12.0%)	
**Profession**				0.352
Professional	0 (0.0%)	5 (0.9%)	5 (0.8%)	
Secretarial	0 (0.0%)	0 (0.0%)	0 (0.0%)	
Trader	26 (32.1%)	207 (36.1%)	233 (35.6%)	
Seamstress	4 (4.9%)	39 (6.8%)	43 (6.6%)	
Farmer	50 (61.7%)	321 (56.0%)	371 (56.7%)	
Other	1 (1.2%)	1 (0.2%)	2 (0.3%)	
**Participant's Education** (years)				0.009
Mean (SD)	8.1 (5.4)	6.5 (5.7)	6.7 (5.7)	
**Wealth Index quintile**				0.744
1 (very poor)	21 (21.4%)	133 (19.6%)	154 (19.8%)	
2	17 (17.3%)	140 (20.6%)	157 (20.2%)	
3	17 (17.3%)	141 (20.7%)	158 (20.3%)	
4	23 (23.5%)	132 (19.4%)	155 (19.9%)	
5 (least poor)	20 (20.4%)	134 (19.7%)	154 (19.8%)	

Table S4: Univariable linear regressions of participant and intervention characteristics on total RANAS score change from the pre to post tests. Positive estimates indicate an increase in the RANAS post-test score.

Term	Estimate	P value
Number of visits from community-based surveillance volunteer	**0.10**	**0.02**
Religion: Non-Christian	-0.77	0.42
Participant's age	0.04	0.50
Participant's years of education	-0.05	0.49

Figure S2: Time series of use over the entire study period, relative to date of enrollment, including imputed values. Dashed line = 6 months.

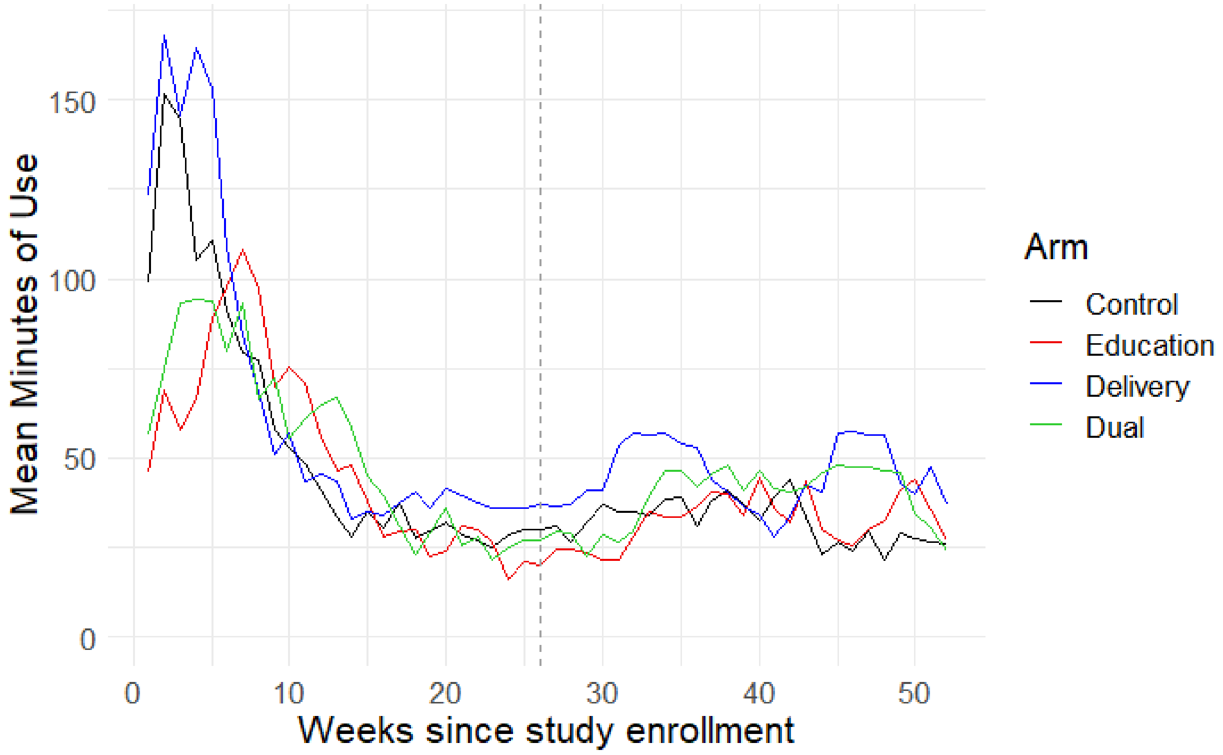

Table S5: Cost of a 14.5 kg cylinder refill at local refilling station.

Month/Year	LPG Price (GH₵)
September 2017	65.00
May 2018	69.00
June 2018	70.00
August 2018	72.00

Table S6: Subgroup analysis of treatment effect via log linear regression with interactions between socio-demographic variable (term) and treatment arm on cooking time in the final 6 months of ELAG. Values = point estimate (95% confidence intervals).

Term	Delivery	Dual	Education
Participant's Age	-0.01 (-0.03-0.01)	-0.02 (-0.04-0.01)	0 (-0.02-0.03)
Asset index	-0.06 (-0.14-0.03)	0.04 (-0.04-0.13)	-0.04 (-0.12-0.04)
Ethnicity (reference = Akan)			
Dagarti	-0.01 (-0.63-0.61)	0.53 (-0.21-1.27)	-0.14 (-0.91-0.63)
Grushi	0.57 (-0.28-1.42)	0.34 (-0.78-1.45)	-0.22 (-1.17-0.74)
Konkomba	-0.36 (-1.16-0.45)	0.05 (-0.76-0.87)	-1 (-1.89--0.11)
Mo	-0.17 (-1.17-0.84)	-0.17 (-1.33-0.98)	-0.39 (-1.53-0.74)
Other	0.23 (-0.54-1.01)	0.28 (-0.47-1.03)	-0.17 (-1.03-0.7)
Household Size (reference = 2-5 people)			
HHSize6-10 people	0.25 (-0.13-0.62)	0.16 (-0.23-0.55)	0.25 (-0.14-0.65)
HHSize>10 people	0.26 (-0.33-0.86)	0.54 (-0.11-1.19)	0.36 (-0.22-0.95)
Relationship Score	0 (-0.04-0.03)	0 (-0.04-0.03)	0.01 (-0.03-0.04)
Religion (reference = Christian)			
Non-Christian	0.13 (-0.27-0.52)	-0.15 (-0.61-0.31)	-0.04 (-0.45-0.37)
Profession (reference = Professional)			
Farmer/Domestic Worker	-0.09 (-0.63-0.44)	-1 (-3.12-1.13)	-0.79 (-2.54-0.96)
Secretarial	-0.14 (-0.69-0.41)	-1.44 (-3.57-0.69)	-0.58 (-2.34-1.18)
Trader	0.69 (-0.21-1.6)	-1.12 (-3.43-1.19)	0.1 (-1.82-2.01)
Unknown/No Response	NA	-0.68 (-2.86-1.5)	-0.74 (-2.53-1.04)
